# Spatial Distribution and Quantification of Mammographic Breast Density, and Its Correlation with BI-RADS Using an Image Segmentation Method

**DOI:** 10.3390/life11060516

**Published:** 2021-06-03

**Authors:** Yi Ling Eileen Goh, Zhen Yu Lee, Christopher Lai

**Affiliations:** Health and Social Sciences, Singapore Institute of Technology, 10 Dover Drive, Singapore 138683, Singapore; 1701964@sit.singaporetech.edu.sg (Y.L.E.G.); 1701578@sit.singaporetech.edu.sg (Z.Y.L.)

**Keywords:** mammographic images, mammographic breast density, image segmentation, spatial autocorrelation, overall percentage density, BI-RADS

## Abstract

(1) Background: Mammographic breast density (MBD) and older age are classical breast cancer risk factors. Normally, MBDs are not evenly distributed in the breast, with different women having different spatial distribution and clustering patterns. The presence of MBDs makes tumors and other lesions challenging to be identified in mammograms. The objectives of this study were: (i) to quantify the amount of MBDs—in the whole (overall), different sub-regions, and different zones of the breast using an image segmentation method; (ii) to investigate the spatial distribution patterns of MBD in different sub-regions of the breast. (2) Methods: The image segmentation method was used to quantify the overall amount of MBDs in the whole breast (overall percentage density (PD)), in 48 sub-regions (regional PDs), and three different zones (zonal PDs) of the whole breast, and the results of the amount of MBDs in 48 sub-regional PDs were further analyzed to determine its spatial distribution pattern in the breast using Moran’s I values (spatial autocorrelation). (3) Results: The overall PD showed a negative correlation with age (*p* = 0.008); the younger women tended to have denser breasts (higher overall PD in breasts). We also found a higher proportion (*p* < 0.001) of positive autocorrelation pattern in the less dense breast group than in the denser breast group, suggesting that MBDs in the less dense breasts tend to be clustered together. Moreover, we also observed that MBDs in the mature women (<65 years old) tended to be clustered in the middle zone, while in older women (>64 years old) they tended to be clustered in both the posterior and middle zones. (4) Conclusions: There is an inverse relationship between the amount of MBD (overall PD in the breast) and age, and a different clustering pattern of MBDs between the older and mature women.

## 1. Introduction

According to the World Health Organization (WHO), female breast cancer has topped as the most diagnosed cancer worldwide [1,2]. Mammographic breast density (MBD) is increasingly known to be a strong, independent risk factor for breast cancer; MBD is the ratio of radiodense fibroglandular tissue, which is fibrous connective tissue (the stroma) and the functional (or glandular) epithelial cells that line breast ducts, to radiolucent adipose tissue [3,4]. Adipose tissues, which have a much lower X-ray attenuation coefficient than fibroglandular tissues, may be protective against breast carcinogenesis [5]. However, the X-ray attenuation coefficient of breast tumors is like that of fibroglandular tissue; thus, breast tumors are also shown as areas of radiodensity.

High MBD was associated with a 1.8- to 6.0-fold increase in breast cancer risk [6,7,8,9]. Although women of younger age have higher MBD than older women, breast cancer occurrence generally increases with age, with older women having the highest incidence rates of median diagnosis age at 62 and with the highest probability of being diagnosed with invasive breast cancer for women in their 70s [10]. 

Looking at the relationship between MBD, age, and breast cancer, younger women should have higher cancer incidence rates, since their MBD is also higher. However, other factors could also affect the risk of breast cancer, such as geographic location, age at menarche, menopause age, hormonal replacement therapy, family history of breast cancer, body mass index (BMI), number of menstrual cycles, and socioeconomic status [11,12]. Katz (2019) found that the increase in menstrual cycles increased the risk of breast cancer. Breast cancer screening is more effective in older women as MBD decreases due to post-menopause breast glandular alterations than higher MBD in younger women, which can mask lesions, thus affecting the diagnostic sensitivity of mammography [3,13]. Among all the risk factors listed in the literature, as mentioned earlier, the age factor has the highest relative risk for breast cancer with the elderly women group at risk [12].

Wolfe et al. (1976) were the first to establish an association of variation in MBD with the risk of breast cancer; additionally, they were the first to come up with the well-known Wolfe breast density classification—N1 (predominantly fat), P1, and P2 (ductal prominence in “less than one-fourth or more than one-fourth,” respectively, of the breast), and DY (extensive “dysplasia”) [14]. Currently, there are five principal methods to assess MBD: Wolfe classification, Breast Imaging Reporting and Data System (BI-RADS), planimetry, and computer-assisted thresholding; the former three methods are qualitative, while the latter two are quantitative methods for visual estimation [15]. Many factors are associated with higher MBD, including younger age, higher body mass index (BMI), lower parity, higher first-child postpartum age, higher education level, and family history of breast cancer [13].

Image segmentation is defined as “separating the image into similar constituent parts, including identifying and partitioning regions of interests” [16]. Image segmentation of mammographic images provides more information at the local level than breast classification by segmenting into radiolucent adipose and radiodense fibroglandular tissues, and automated thresholding algorithms can overcome observer bias in choosing threshold values subjectively to achieve optimum segmentation objectively [17,18]. The thresholding method can identify and extract the region of interest, such as the foreground (fibroglandular tissue) from its background (adipose tissue) based on the texture (grey-level distribution) in image areas. An example is based on entropy distribution of the grey levels in mammographic images, which after the ratio value between the foreground and background is quantified after thresholding [19,20]. The ratio of the radiodense (white) area over the entire breast area is known as the quantified MBD. 

The spatial distribution pattern of MBD can depend on various factors, such as physiological, genetic, environmental, and pathological. Pereira et al. (2009) studied the spatial distribution of breasts in women and concluded that age is not a greater factor in the degree of clustering of high-density areas in women’s breasts. However, they did an eight-year longitudinal study on women aged between 39–41, a narrow age range of women below 50 years old [21]. Another research by Lai and Law (2015) studied the spatial distribution patterns of MBD in 50 Chinese women aged 40 to 60 at the entry mammographic examination. They concluded that the MBD is spatially autocorrelated together in different regions for Chinese women of different breast size; clustering occurs in the anterior part of the breast in those with smaller breast size, while those with a larger breast size tend to have their MBD clustered near the posterior part of the breast [22]. From the comparison of entry and exit mammograms (after three years since the entry), MBD clustering decreased with reduced Moran’s I values, and a tendency towards a more dispersed pattern was observed.

The objectives of this current work are: (i) to quantify the amount of MBD in terms of overall PD and over different regions/zones of the breast using the image segmentation method; and (ii) to investigate the spatial distribution patterns of MBD within the breast of segmented mammographic images.

## 2. Materials and Methods

### 2.1. Database and Sample Population

The study worked on normal screening digitized film-screen mammographic images from the Digital Database for Screening Mammography (DDSM) based on women from the USA [23]. Each case (as per woman) comes in four projections—left mediolateral oblique (LMLO), right MLO (RMLO), left craniocaudal (LCC), and RCC. Each case information consists of the patient’s age and MBD category (density group) according to the BI-RADS density classification [24]. Based on the median breast cancer diagnosis age of 62 from the American Cancer Society (2019), this study divided the cases into two age groups—mature women (35–64 years old) and older women (65–90 years old). 

A total of 529 LMLO mammographic images of normal screening cases were extracted from DDSM with exclusion criteria of benign cases and 1 case in which a pacemaker overlapped with the radiodense areas of the breast. The rationale for a single-sided projection (i.e., LMLO) selection is that the spatial distribution and amount of MBD are similar for both breasts [18], and the MLO view allows for the visualization of the maximum coverage of a breast until the pectoral muscles in a single projection. 

### 2.2. Data Pre-Processing

**Figure 1:**
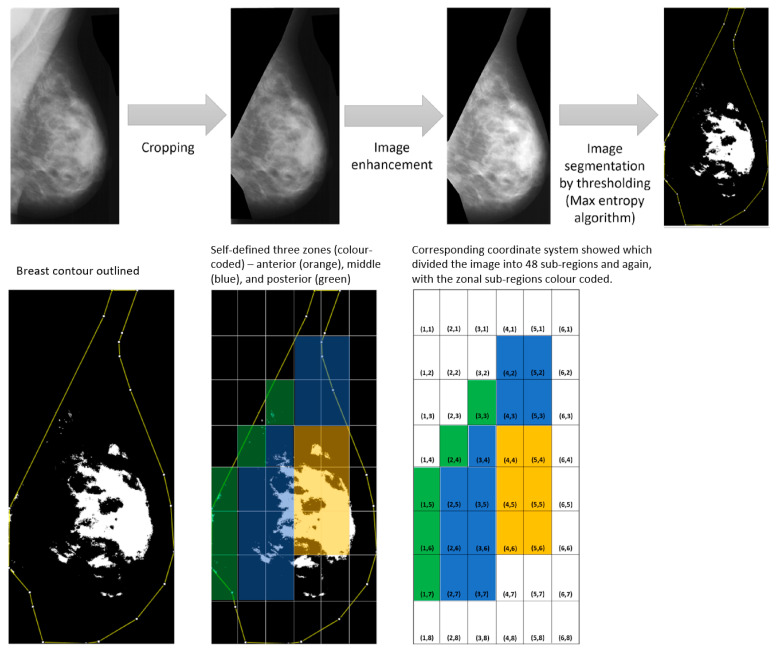
Pre- and post-image segmentation processes of extracted LMLO mammographic images.

Extracted anonymized and digitized mammographic images (converted from lossless JPEG format to PNG format) were processed using the NIH ImageJ program (Version 1.51, Rasband, US National Institute of Health, Bethesda, MD, USA), to define the pectoral muscle and the breast contour, while the area outside of the defined region of interest (ROI) was cropped out (Figure 1). This procedure was to avoid the miscalculation of the radiodensity by considering radiodense objects outside of the ROI. A contrast enhancement function was applied to the images to better outline the breast borders, because the X-ray attenuation of the breast near the skin borders is the lowest due to reduced breast tissue thickness.

### 2.3. Breast Tissue Segmentation and Quantification of Overall Percentage MBD in Segmented Images

Segmentation of the cropped and pre-processed LMLO images (Figure 1) into radiodense (highly X-ray attenuating fibroglandular tissue) and non-dense (low X-ray attenuating adipose tissue) areas used the Maximum Entropy Threshold algorithm [25] in the ImageJ program. 

After defining the contour of the breast in the ImageJ program, the overall percentage mammographic density in the whole breast (overall PD) in terms of radiodensity could be quantified by the ratio of the white area (radiodensity) to defined breast area (radiodensity and radiolucency areas) × 100%.

### 2.4. Division into 48 Sub-Regions and Self-Defined Three Zones of the Breast

After image segmentation (Figure 1), each segmented image was divided into 48 equally sized rectangular sub-regions (8 × 6 matrix of six columns and eight rows with coordinates) by TileMage Image Splitter software (Version 2.0.1), OrangeBright, Israel. Individual sub-regional MBD (regional PD) was quantified and tabulated. Then, the three zones of the breast—posterior, middle, and anterior—were self-defined, as shown in Figure 1. The mean zonal percentage mammographic density (zonal PD) was calculated by averaging the regional PDs within each of the self-defined zones.

### 2.5. Spatial Autocorrelation

The regional PD values were used to estimate the spatial autocorrelation (using Moran’s I equation) of the 48 sub-regions (per breast):

(1) $$Moran’s\;I=\frac{N}{\sum_{i}\sum_{i}w_{\mathrm{ij}}}\frac{\sum_{i}\sum_{j}w_{\mathrm{ij}}\left(x_{i}-\bar{x}\right)\left(x_{j}-\bar{x}\right)}{\sum_{i}{\left(x_{i}-\bar{x}\right)}^{2}}$$

where the weighting factor *w_ij_* is defined as 1/d^2^, and d refers to the distance between the midpoints of adjacent two sub-regions [22,26]. Moran’s I value ranges from −1 to 1, whereby a positive autocorrelation (>0) is a clustered spatial distribution pattern of MBD, no autocorrelation (=0) is a random pattern, and a negative autocorrelation (<0) is a scattered spatial distribution pattern of MBD [21].

### 2.6. Statistical Methods

IBM SPSS Statistics (Version 26) software, IBM Corp., Armonk, NY, USA, was used, and all statistical analyses were done at a 95% confidence interval. For continuous outcome variables, such as age, overall segmented MBD, and mean zonal PDs, a Shapiro–Wilk test was applied to test for normality—if a distribution was normal, *p* > 0.05. Non-parametric tests were done for all statistical analyses due to the non-normality of the continuous distributions studied (except for the mean age comparison) and the nominal nature of the other outcome variables.

#### 2.6.1. Age, MBD, Overall PD, and Zonal PDs

The independent *t*-test compared the mean age in the less dense breasts group and denser breasts group, while the Pearson’s correlation test was used to check for any association of overall PD with age. Median posterior, middle, and anterior zonal PDs were compared across the four BI-RADS density groups using Kruskal–Wallis tests; Friedman tests were applied within each zone (anterior and middle) to make pair-wise comparisons of median zonal PD of different BI-RADS density groups.

#### 2.6.2. Analysis of Spatial Autocorrelation Data

The spatial distribution pattern of MBD was tabulated for the two age groups of women (mature and older women). Median Moran’s I value from each age group was then computed to indicate either a clustered, random, or scattered pattern. The spatial autocorrelation in the four different BI-RADS density categories was compared using the Chi-square test.

#### 2.6.3. Clustering of Breast Tissue Density

The clustering locations of MBD between two age groups of women (mature women versus older women) were studied. Clustering of MBD can occur at different zones of the breast—anterior, middle, or posterior (Figure 1). A Mann–Whitney U test was used to check for any significant statistical difference in the medians of three zonal PD within these two age groups.

## 3. Results

### 3.1. Demographics of Women in This Study

**Figure 2:**
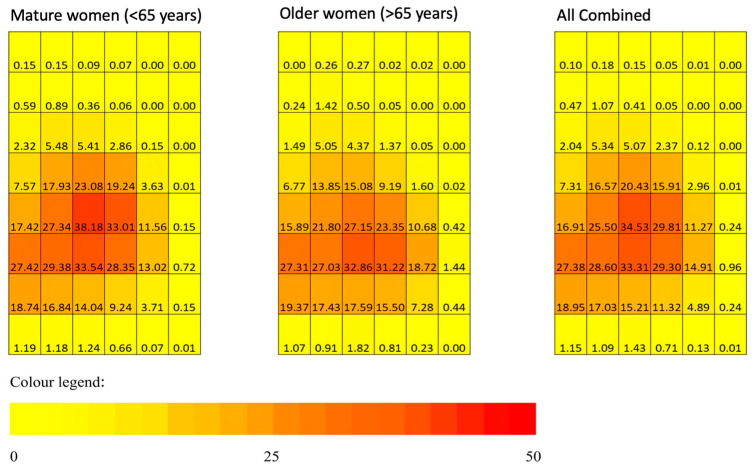
Mean regional PD in each of the 48 sub-regions stratified by age groups (mature vs. older women). The numerical value inside the coordinate system corresponds to the amount of MBD at the individual sub-region. The amount of MBD determines the MBD heatmaps with different hues (from yellow to orange to reddish-orange to red) of each sub-region after segmentation. Yellow color—none or minimal amount of MBD; orange color—about 25% of MBD; red color—equal to or more than 50% of MBD.

The heatmaps in Figure 2 showing individual regional PD in each of the 48 sub-regions stratified by age groups (mature vs. older women). It shows that mature women had more sub-regions containing higher amounts of MBD. When the whole population were further divided into BI-RADS density groups for analysis, the mean age of 56.7 was statistically significantly (*p* < 0.001) younger in the denser breasts group (combining BI-RADS groups C and D) when compared to the mean age of 61.8 in the less dense breasts group (combining BI-RADS groups A and B), (Table 1).

### 3.2. Age and Quantification of the Segmented Overall Amount of MBD (Overall PD)

**Figure 3:**
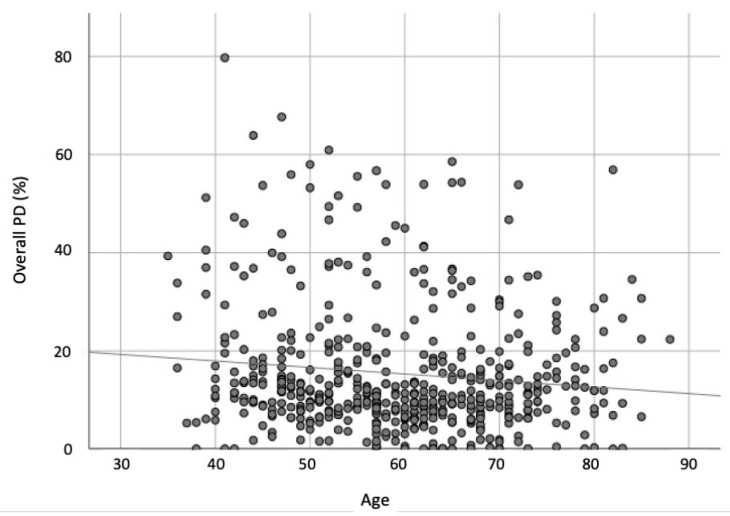
Simple scatter plot of overall PD (%) by age (year).

Using Pearson’s correlation test, the overall PD of segmented mammographic images showed a statistically significant negative correlation *p* = 0.008 (*p* < 0.05) with the age of women, i.e., as the age of women increased, the overall PD had a decreasing trend (Figure 3).

### 3.3. Spatial Distribution and BI-RADS Density Groups

The spatial distribution pattern was determined by Moran’s I values, in which a positive autocorrelation indicates a clustered pattern, while a negative autocorrelation indicates a scattered pattern. For the less dense breasts groups (BI-RADS density A and B; *p* < 0.001 for both groups), a higher proportion (74.0% and 66.1%) of women had a clustered pattern of MBD (positive autocorrelation), whereas denser breasts groups (BI-RADS density C and D; *p* < 0.001 and *p* = 0.030, respectively) had a higher proportion (53.8% and 50.8%) of women with a scattered pattern of MBD (negative autocorrelation). Overall, there was a statistically significant difference (*p* < 0.001) in the spatial autocorrelation across the four BI-RADS density groups (Table 2). 

### 3.4. Zonal PDs and BI-RADS Density Groups

**Figure 4:**
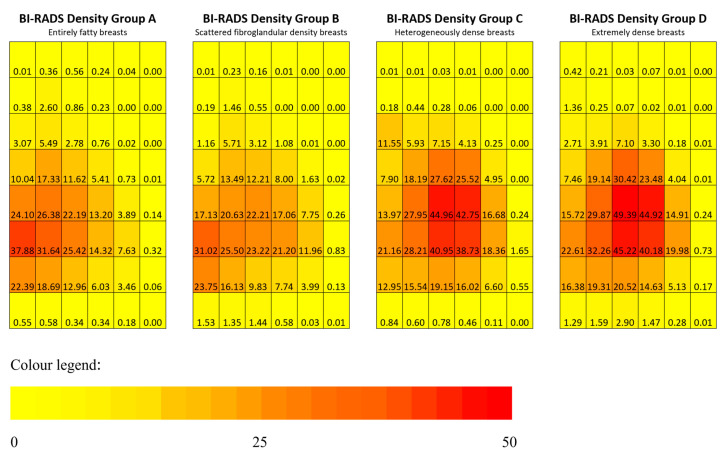
Mean individual sub-region MBD stratified by BI-RADS density groups. The numerical value inside the coordinate system corresponds to the amount of MBD at the individual sub-region. The amount of MBD determines the MBD heatmaps with different hues (from yellow to orange to reddish-orange to red) of each sub-region after segmentation. Yellow color—none or minimal amount of MBD; orange color—about 25% of MBD; red color—equal to or more than 50% of MBD.

**Figure 5:**
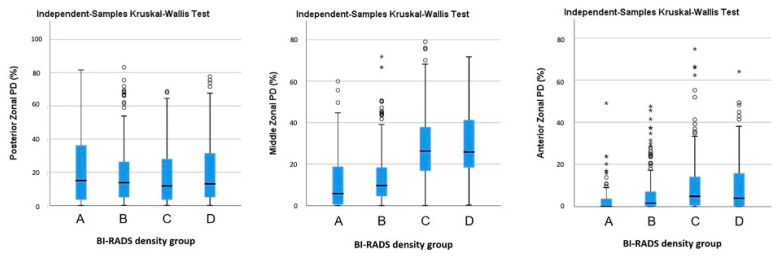
Posterior, middle, and anterior zonal PDs across the four BI-RADS density groups. Comparison across four BI-RADS density groups: *p* = 0.761 for posterior median zonal PD; *p* < 0.001 was statistically significant for both the middle and anterior median zonal PDs. The outliers and extremes are expressed as circles and asterisks, representatively.

In Figure 4 (heatmaps of MBD), the denser breasts groups (C and D) had higher mean MBD in the middle zone, while the less dense breasts groups (A and B) had a more even distribution of MBD in the posterior and middle zones, but with much lower MBD values. Using Kruskal–Wallis tests to compare the median posterior, middle, and anterior zonal PDs across the four BI-RADS density groups, the middle and anterior median zonal PDs were found to be statistically significantly different, with both having *p* < 0.001, while *p* = 0.761 for the posterior median zonal PD (Figure 5). Friedman tests (Table 3) were applied within each zone (anterior and middle zones) to make pair-wise comparisons of the BI-RADS density groups. Within the middle zones, the median zonal PDs are statistically different (*p* < 0.05) between A–C, A–D, B–C, and B–D pairs, while within the anterior zones, the median zonal PDs were statistically different (*p* < 0.05) between A–B, A–C, A–D, and B–D. 

### 3.5. Spatial Clustering Location and Age Group

**Figure 6:**
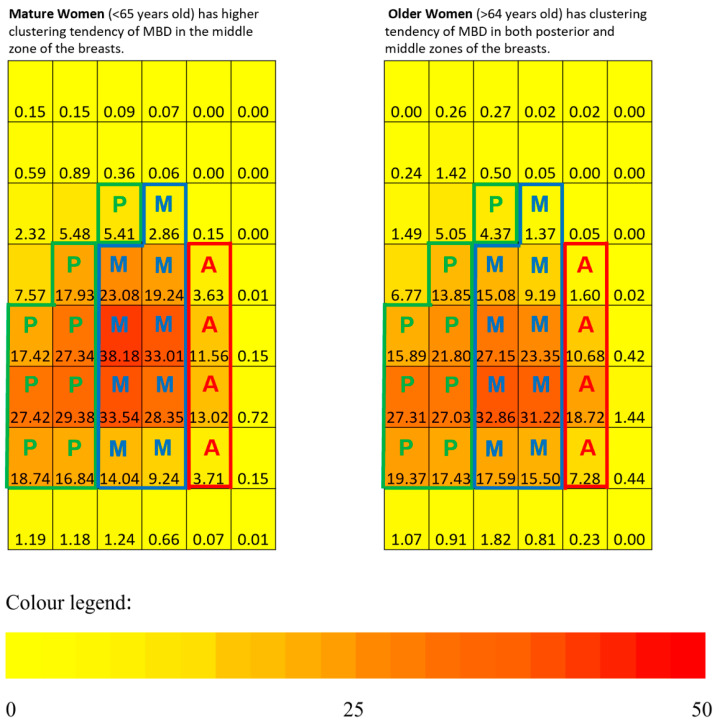
Comparison of mean individual regional PD between the older and mature women groups. The numerical value inside the coordinate system corresponds to the amount of MBD at the individual sub-region. The amount of MBD determines the MBD heatmaps with different hues (from yellow to orange to reddish-orange to red) of each sub-region after segmentation. Yellow color—none or minimal amount of MBD; orange color—about 25% of MBD; red color—equal to or more than 50% of MBD. P—posterior zone; M—middle zone; A—anterior zone.

The overall median zonal PD of the anterior, middle, and posterior zones are 2.3%, 17.4%, and 13.6%, respectively, with *p* < 0.001 comparing the overall median PD across the three zones. Table 4 further compared the median zonal percentage density (PD) across the mature and older women groups, and the mean individual regional PD was shown in Figure 6. It was found that only the middle zonal PD are statistically significantly different (*p* = 0.046), with mature women having higher middle zonal PD. In addition, pairwise comparisons of median zonal PD within the older and mature women groups were made. There were statistically significant differences in zonal PD in the older women group between anterior–middle and anterior–posterior zones (*p* < 0.001), whereas in the mature women group, statistically significant differences of PD existed between all pairs, i.e., anterior–posterior, anterior–middle, and posterior–middle zones. 

## 4. Discussion

### 4.1. Age and Segmented Overall Amount of MBD (Overall PD)

Previous studies by Boyd et al. (2010) and Li et al. (2005) identified increasing age to be one of the factors to decrease the population average MBD [15,27]. From this study, our younger age group—the mature women (<65 years old)—demonstrated that this age group has denser breasts than the older women (>64 years old), and the overall PD of segmented MBD amount in the breast showed a negative correlation (*p* = 0.008) with age. This result emphasized that younger women are more likely to have dense breasts and that breast tissue becomes less dense as women age. This inverse relationship between MBD and age is because as women age and go through menopause, reductions in stromal and epithelial breast tissues are reflected as decreased average MBD [3,15,27]. Another six-year longitudinal study (with three data points; two-year intervals) conducted by Oliver et al. (2015) confirmed the decreasing trend of MBD in breasts over time, and they also noticed the decrease in the amount of MBD associated with the initial amount of MBD in women—particularly the densest breast group (Group D) with a slower decrease in the MBD [18].

### 4.2. Comparison of the Spatial Distribution of MBD and Zonal PDs with the Different BI-RADS Density Groups

When comparing the four BI-RADS density groups, there was a statistically significant (*p* < 0.001) higher proportion of positive autocorrelation (having a clustering of MBD) observed in the less dense breast groups (Group A: 74.0%; Group B: 66.1%) than those in the denser breast groups (Group C: 46.2%; Group D: 49.2%). This finding suggested that MBD in the less dense breasts tends to be more clustered together, while MBD in denser breasts is more dispersed than the less dense breasts. One possible explanation could be that less dense breasts have smaller areas of fibroglandular tissue and more adipose tissue. After image segmentation, the radiodense areas (fibroglandular tissue) clustered mainly in the middle zones and could be differentiated from larger surrounding radiolucent adipose areas. The MBD clustering location would affect the median zonal PDs of the posterior, middle, and anterior zones. Upon comparison of the median zonal PDs in the posterior zone across the four BI-RADS density groups, there was no statistical difference in the median posterior zonal PD. Meanwhile, there were statistically significant differences in the median zonal PDs in the middle and anterior zones, showing changes in the amount of MBD clustering in these two zones of the breast.

### 4.3. Comparison of Spatial Clustering Locations with Age Groups

This study demonstrated the clustering spanning over the middle zones for mature women (Figure 6), similar to a previous study by Pereira et al. (2009), which had women with a similar ethnicity (Caucasian women) and age group (39–49 years old versus mature women’s age range of 35–64 years old) to this study; the middle zone of the breast consists mainly of mammary gland lobules. Contrary to our results, this study showed a new finding with older women, demonstrating clustering of MBD in both the posterior and middle zones, possibly due to a greater decrease of MBD with age over the middle zones. This finding was supported by the greater middle zonal PDs in the mature women group than the older women group, and there was no statistically significant difference in the posterior–middle zones within the breasts of those in the older women group. Thus, this could also suggest that MBD decreases in the middle zone of the breast over time due to mammary gland lobules undergoing regression with increasing age [28].

### 4.4. Limitations of this Study

This study did not consider the thickness and volume of the breast in the determination of MBD due to the use of the DDSM, which is a very dated dataset in which the original forms were screen-films which later become digitized. The information accompanying the mammographic images was limited as other demographics details were absent, such as ethnicity, parity, usage of hormonal therapy, and menopausal age. These factors could also be potential confounders affecting MBD and spatial distribution patterns. The digitization process of screen-film mammographic images may unintentionally create speck artefacts on the final images and confuse them with MBD radiodensity. This research was a retrospective cross-sectional population study; thus, the longitudinal effects of MBD with age could not be explored.

### 4.5. Proposed Future Recommendations

Other thresholding algorithms, such as Otsu, can be used to compare the effectiveness of image segmentation and quantification of MBD. This current work suggested the decrease in middle zonal PDs in older women was that mammary gland lobules, mainly located in the middle regions of the breasts, regress as women age. With this knowledge, further longitudinal studies could investigate if the decrease in MBD over time can affect the spatial distribution and autocorrelation patterns of MBD in women, such as the possible shift in zonal clustering location of MBD over time or the change in spatial autocorrelation patterns as women age.

## 5. Conclusions

This study showed that mature women (<65 years old) have denser breasts than older women (>64 years old); age was, indeed, a factor affecting the amount of MBD, as evidenced by the mean age of 56.7 in the denser breasts and 61.8 for the less dense breasts groups. There was a general trend of decreasing overall PD with increasing age, i.e., the overall PD of the segmented mammographic images had a negative correlation with age. As for the spatial distribution patterns of MBD, we used Moran’s I values to determine the spatial autocorrelation and found that there was a more significant proportion of positive autocorrelation in the less dense breasts group than in the denser breasts group; this suggested that MBD in the less dense breasts tends to have clustered spatial distribution patterns. By considering the finding from median zonal PDs, mature women tended to have a clustering of MBD in the middle zone, while older women tended to have a clustering of MBD in both posterior and middle zones.

The image segmentation and quantification of MBD can help radiologists to identify extremely dense breasts and highly clustered MBD location areas associated with carcinogenesis.

## Figures and Tables

**Table 1:** The distribution of MBD by age group and BI-RADS density groups.

		Breast Density				
		Less Dense Breasts		Denser Breasts		
	BI-RADS Density Group	A	B	C	D	Total
Number of Cases by Age group	Mature Women	39	118	101	95	353
	*n* (%)	(53.4)	(61.5)	(69.7)	(80.5)	(66.9)
	Older Women	34	74	44	23	175
	*n* (%)	(46.6)	(38.5)	(30.3)	(19.5)	(33.1)
	All age combined	73	192	145	118	528
	*n* (%)	(13.8)	(36.4)	(27.5)	(22.3)	(100)
	Mean Age (year ± SD)	61.8 ± 10.5		56.7 ± 11.3		Independent *t*-test, *p* < 0.001

**Table 2:** Spatial distribution patterns versus different BI-RADS groups.

Chi-Square Test	Moran’s I (Counts)		*p*-Value
	Negative Autocorrelation Scattered Pattern	Positive Autocorrelation Clustered Pattern	
BI-RADS density group A *Entirely fatty breasts* (*n* = 73)	19 (26.0%)	54 (74.0%)	<0.001
BI-RADS density group B *Scattered fibroglandular density breasts* (*n* = 192)	65 (33.9%)	127 (66.1%)	<0.001
BI-RADS density group C *Heterogeneously dense breasts* (*n* = 145)	78 (53.8%)	67 (46.2%)	<0.001
BI-RADS density group D *Extremely dense breasts* (*n* = 118)	60 (50.8%)	58 (49.2%)	0.030
Total (*n* = 528)	222 (42.0%)	306 (58.0%)	<0.001

**Table 3:** Pairwise comparisons of median anterior and middle zonal PDs in different BI-RADS density groups.

Friedman Tests	*p*-Values	
Pair-Wise Comparison between BI-RADS Desnity Groups	Middle Zonal PD	Anterior Zonal PD
A–B	0.302	0.001
A–C	<0.001	<0.001
A–D	<0.001	<0.001
B–C	<0.001	0.056
B–D	<0.001	0.003
C–D	0.457	0.382

**Table 4:** Median zonal PD of three zones stratified by age group and post-hoc pairwise comparison of three zonal PD within the age group to locate MBD clustering.

Mann–Whitney U Test	Median Zonal PD (in MBD %, Standard Deviation in Parentheses)		*p*-Value
	Mature Women (<65 Years Old)	Older Women (>64 Years Old)	
Posterior Zonal PD	14.0 (19.5)	12.8 (18.7)	0.221
Middle Zonal PD	17.6 (18.1)	16.4 (17.1)	0.046
Anterior Zonal PD	2.4 (10.7)	2.0 (14.4)	0.887
Post-hoc pair-wise comparisons of Zonal PD in older and mature women age groups (*p*-value)			
	Anterior–Posterior	Anterior–Middle	Posterior–Middle
Older Women	<0.001	<0.001	0.855
Mature Women	<0.001	<0.001	0.005

## Data Availability

Not applicable.
